# Pica in Sickle Cell Disease: A Systematic Review and Meta‐Analysis

**DOI:** 10.1155/anem/5589148

**Published:** 2026-08-19

**Authors:** Melanie Bruinooge, Marc J. M. Oubrie, Maarten F. M. Engel, Sabine E. Herrman-Mous, Marjon H. Cnossen

**Affiliations:** ^1^ Department of Paediatric Haematology and Oncology, Erasmus University Medical Center–Sophia Children’s Hospital, Rotterdam, the Netherlands; ^2^ Medical Library, Erasmus University Medical Center, Rotterdam, the Netherlands, erasmusmc.nl; ^3^ Department of Child and Adolescent Psychiatry/Psychology, Erasmus University Medical Center–Sophia Children’s Hospital, Rotterdam, the Netherlands

**Keywords:** characteristics, pica, prevalence, sickle cell disease, systematic review

## Abstract

Pica, the persistent ingestion of non‐nutritive, non‐food substances for ≥ 1 month at a developmentally inappropriate age, is regularly observed but understudied in sickle cell disease (SCD). Its prevalence and risk factors are unclear. This systematic review aims to estimate pica prevalence and synthesize evidence on associated characteristics and correlates in SCD. We searched Embase, MEDLINE ALL, Web of Science, Cochrane CENTRAL, PsycINFO and Google Scholar up to 31 October 2025, for studies reporting original data on the prevalence and characteristics of pica in individuals ≥ 2 years with SCD. Risk of bias was assessed using a modified Newcastle–Ottawa Scale. Meta‐analysis of prevalence used random‐effects models. Data on substances consumed were pooled. Literature on potential correlates was narratively summarized across biological, psychological and social domains. Ten studies (1585 participants) from North America (*n* = 6), Africa (*n* = 1), Europe (*n* = 1), the Middle East (*n* = 1) and South America (*n* = 1) were included, with sample sizes ranging from 55 to 395. The pooled prevalence of pica was 34% (95% CI: 24%–46%, I^2^ = 94%). Across four studies, the most commonly ingested materials were clay/dirt (*n* = 110, 39.3%), paper (*n* = 60, 21.4%) and fabric (*n* = 29, 10.4%). Narrative synthesis identified younger age, lower bodyweight, lower haemoglobin levels, higher impulsivity, lower socioeconomic status, lower family satisfaction and a positive family history as potential correlates of pica in SCD. Pica in SCD is highly prevalent. Psychosocial determinants may play a larger role than previously anticipated, alongside biological and disease‐related contributors. However, current evidence is limited by small sample sizes, inconsistent findings, and lack of validated assessment tools. To advance understanding of the aetiology of pica, future longitudinal studies integrating biological, psychological, social and neuroimaging measures are needed.

## 1. Introduction

Pica is defined as the persistent ingestion of non‐nutritive, non‐food substances for > 1 month at a developmentally inappropriate age (typically > 18 months) [1]. Commonly ingested materials include clay/soil, starch and fabric, though a variety of other substances can also be consumed [2].

The prevalence of pica in the general population is estimated to be around 2% at the age of 36 months in healthy children [2], while studies in children with sickle cell disease (SCD) report widely varying prevalence rates of between 9% [3] and 66% [4]. Despite its reported prevalence and the associated burden for patients and their families, pica in SCD is strikingly understudied, especially in adults. Importantly, pica may lead to complications such as electrolyte imbalance, nutritional deficiencies, poisoning from exposure to toxic contaminants (e.g., lead) and bowel obstruction sometimes leading to acute surgery [5–8]. Beyond these direct effects, pica may complicate the clinical picture in more indirect ways. Abdominal pain caused by pica (e.g., bezoars, constipation or gastrointestinal irritation) [9] can be confused with abdominal pain during a vaso‐occlusive crisis (VOC), potentially delaying appropriate management. Moreover, electrolyte disturbances may precipitate ECG abnormalities or cardiac arrhythmias [9], which can destabilize a cardiovascular system already compromised by cardiomegaly, diastolic dysfunction, and chronic anaemia in SCD [10]. Toxic exposure may exacerbate hepatic or renal injury [11]. Additionally, pica may increase infection risk [9] in patients already having diminished immunological defences due to functional asplenia [10]. These considerations underscore the importance of timely identifying pica behaviour and better understanding of underlying mechanisms and best therapeutic approaches.

In the general population, pica has been linked to biological factors (malnutrition, iron and zinc deficiency, and pregnancy) [9], psychological factors (obsessive‐compulsive disorder, autism spectrum disorder and intellectual developmental disorders) [12] and social factors (low socioeconomic status (SES), cultural factors and positive family history). Rodrigues et al. have proposed a biopsychosocial model for pica in SCD, highlighting three interconnected domains: biological (malnutrition, iron/zinc deficiencies, haemolytic anaemia, body mass index (BMI) and disease severity), psychological (dysfunctional eating and emotional impulsivity) and social (family stress, food insecurity, positive family history) [13, 14].

Most studies in SCD have focused on biological causes of pica but with inconsistent findings. For example, while pica in other study populations (e.g., blood donors, pregnant individuals, kidney patients) is consistently linked to anaemia and iron/zinc deficiencies [15–18], these associations could not be confirmed in studies in SCD [3, 4, 19]. Similarly, associations with biomarkers for disease severity, nutritional status, and different haematological measures for anaemia and haemolysis were not confirmed for pica in SCD [3, 4, 19–21]. Given the widely ranging prevalence rates reported and inconsistencies in the available literature with regard to correlates of pica in SCD, there is a need to clarify the prevalence of pica and its associations in SCD.

Therefore, the primary objective of this systematic review and meta‐analysis is to determine the prevalence of pica in SCD. Secondary objectives are to synthesize the available literature on characteristics and correlates of pica in SCD.

## 2. Materials and Methods

### 2.1. Article Retrieval

This systematic review was conducted in adherence to the Preferred Reporting Items for Systematic Reviews and Meta‐Analyses (PRISMA) guidelines. Systematic searches were conducted in Embase, MEDLINE ALL (Ovid), Scopus, Web of Science Core Collection, Cochrane Central Register of Trials (Wiley), PsycINFO (Ovid) and Google Scholar, from inception to 31 October 2025, using key terms including synonyms and Medical Subject Headings related to SCD and pica. The search strategy was designed and executed by an experienced information specialist (M.F.M.E). in collaboration with the primary researcher (M.B.). The full search strategy is provided in Supporting 1. Additional sources included hand‐searching reference lists using backwards citation searching.

### 2.2. Study Selection

After duplicate removal through Covidence as well as manually, titles and abstracts were independently screened by two independent reviewers (M.B. and M.J.M.O). Full texts of potentially eligible studies were retrieved and assessed for eligibility. Discrepancies were resolved through discussion and consensus, with an additional reviewer consulted in case of remaining disagreement (S.E.H.M.).

### 2.3. Inclusion and Exclusion Criteria

Studies were eligible if they reported original data on pica prevalence in SCD patients (any genotype) aged > 2 years. Exclusion criteria were studies in pregnant patients with SCD, studies in individuals with sickle cell trait, non‐English publications, as well as reviews, case reports, comments or editorials.

### 2.4. Data Extraction

A data‐extraction form was developed collaboratively by two researchers (M.B., M.J.O) based on information required to address our research question. We included items essential for characterizing studies, assessing pica prevalence and assessing associations of pica behaviour with correlates. Specifically, extracted data included country, study year, study design, sample size, sex, age, SCD genotype, investigated characteristics and correlates (e.g., demographics, treatments, comorbidities, laboratory values), and main findings. Data were extracted by one reviewer and cross‐checked by a second using a standardized data extraction form. If both data on current pica and lifetime pica were provided, data on current pica was extracted and used for meta‐analysis.

### 2.5. Quality Assessment

Quality assessment was conducted by two independent reviewers (M.B. and M.J.M.O) using a modified version of the Newcastle–Ottawa Scale (NOS) for non‐randomized studies (Supporting 2). Conflicts were resolved through discussion and consensus, with an additional reviewer consulted in case of remaining disagreement (S.E.H.M.).

### 2.6. Data Synthesis

The meta‐analysis of prevalence was conducted using the metaprop function of the meta package in R version 4.3.2. Prevalence of reported pica was converted into proportions for each individual study. Logit‐transformed proportions were used to calculate effect sizes. Subsequently, random‐effects models (REMs) were fitted to the logit‐transformed proportions using the inverse variance method. Pooled proportions were backtransformed to percentages with 95% confidence intervals (CIs) for interpretation. Heterogeneity was assessed using I^2^ and Cochran’s Q test and illustrated through forest plots. An I^2^ value of 0%–25%, 26%–50%, 51%–75%, and 76%–100% indicated low, moderate, substantial and high heterogeneity, respectively. Significant heterogeneity was indicated by *p* < 0.10 for Cochran’s Q. Subgroup analysis was conducted based on the geographical region and study design. For substances consumed, patient‐level data were pooled, excluding items that were not both non‐nutritive and non‐food (e.g., ice). In the narrative synthesis of literature on correlates of pica in SCD, findings were summarized per individual correlate and grouped into three domains: biological, psychological and social, with extracted measures (means, proportions, ORs, 95% CIs, *p*‐values) reported as in the original studies. Variables were identified as possibly correlated with pica if more than 50% of the studies that conducted statistical comparisons reported a statistically significant association or difference between groups.

## 3. Results

### 3.1. Search Results and Characteristics of Eligible Studies

**FIGURE 1:**
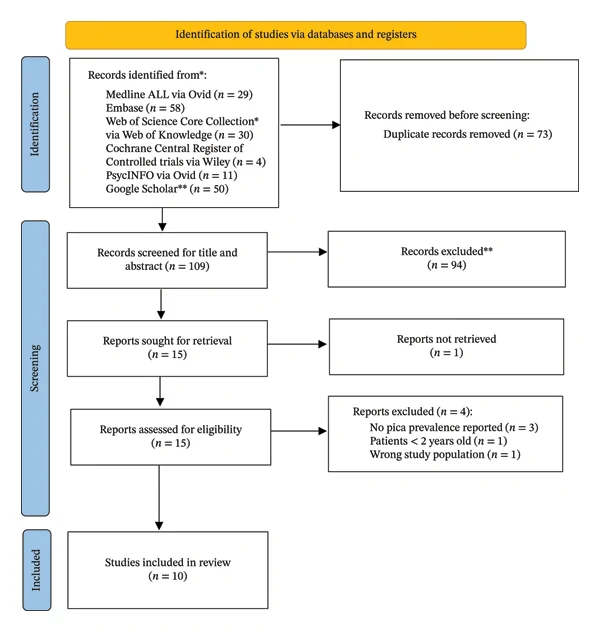
Flowchart of the study selection process. Flow diagram illustrating the selection process of studies included in this systematic review, following PRISMA guidelines. ^∗^Science citation index expanded (1975–present); Social sciences citation index (1975–present); arts & humanities citation index (1975–present); conference proceedings citation index‐science (1990–present); conference proceedings citation index‐social science & humanities (1990–present); emerging sources citation index (2005–present). ^∗∗^Google scholar was searched via “publish or perish” to download the results in endnote.

The literature search identified 182 records across all databases. After removal of duplicates, 109 records remained, of which 10 were found eligible after title and abstract screening and full‐text review. A single discrepancy arose during full‐text review; it was resolved through discussion and consensus, resulting in the article’s exclusion. The article selection process is illustrated in Figure 1.

Table S3T1 (Supporting 3) summarizes study characteristics. Studies were conducted in North America (United States, *n* = 6) [3, 13, 20–22], Africa (Sudan, *n* = 1) [4], Europe (Belgium, *n* = 1) [19], South America (Brazil, *n* = 1) [23] and the Middle East (Saudi Arabia, *n* = 1) [24] between 2001 and 2023, with sample sizes ranging between 55 and 395 (median 150), totalling 1585 patients with SCD. Most studies assessed pica cross‐sectionally (*n* = 7) [4, 13, 19, 21, 22, 24], while three used retrospective chart review (*n* = 3) [3, 20]. Only two studies focused on adults [23, 25]. Five studies included patients with various SCD genotypes (e.g., HbSS, HbSβ0, HbSC) [3, 13, 20, 21], two only included HbSS [4, 23], and three studies did not specify the included genotypes [19, 22, 24].

### 3.2. Quality Assessment

Quality assessment of included studies (Table 1) resulted in the following classifications: unsatisfactory (*n* = 4), satisfactory (*n* = 5), good (*n* = 0) and very good quality (*n* = 1). There were no discrepancies between reviewers during quality assessment. None of included studies used validated tools (e.g., PARDI‐interview) to identify pica, but all cross‐sectional studies employed similar questions (such as ‘Does your child eat anything that isn’t food?’) to assess pica behaviour.

**TABLE 1:** Quality assessment.

	Selection				Comparability		Outcome	
Study	Representative of the cases	Sample size justified and satisfactory	Nonresponse rate	Ascertainment of pica	Potential confounders	Assessment of outcomes	Statistical tests	Total (10/10)
Ahmed et al.	1	1	0	1	1	1	0	5
Aloni et al.	1	1	0	2	1	0	1	6
Ivascu et al.	1	1	1	2	1	2	1	9
Khan et al.	0	1	0	0	0	0	1	2
Lemanek et al.	1	1	1	0	1	1	1	6
Reed‐Knight et al.	1	1	0	0	0	1	0	3
Redding‐Lallinger et al.	1	1	0	0	1	0	0	3
Rodrigues et al.	1	1	0	2	1	1	0	6
Teixeira et al.	1	1	1	0	1	1	1	6
Viswanathan et al.	0	1	0	1	1	1	0	4

*Note:* Quality assessment of included studies using a modified Newcastle–Ottawa Scale. The table summarizes the quality ratings for each included study across all assessed domains. Study quality was classified as follows: very good study; 9–10 points, good study; 7–8 points, satisfactory study; 5–6 points, unsatisfactory study; 0–4 points.

### 3.3. Outcomes

#### 3.3.1. Pooled Prevalence of Pica

**FIGURE 2:**
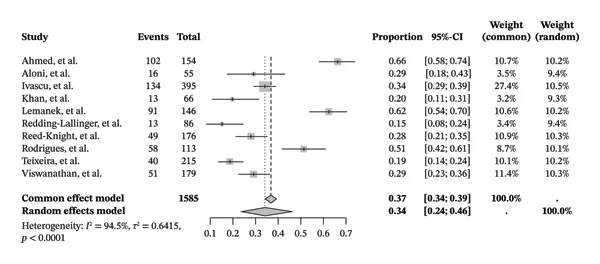
Forest plot pooled proportion of pica. Forest plot displaying proportion estimates of pica from individual studies and the pooled proportion based on common effect and random‐effects models. Squares represent study‐specific proportion with 95% confidence intervals (horizontal lines), with square size proportional to study weight. The diamond represents the overall pooled estimate.

All 10 studies (*n* = 1585 SCD patients) were included in the meta‐analysis, with pica prevalence ranging from 15% to 66% (*n* = 567 affected by pica). The pooled prevalence was 34% (REM 34, 95% CI 24‐46, I^2^ = 94%, *p* < 0.0001) among individuals with SCD. Figure 2 provides a forest plot of the meta‐analysis.

Subgroup analyses were conducted based on the geographical region and study design (Figure S3F1 and S3F2 in Supporting 3, respectively). Subgroup differences for the geographical region based on REMs were found to be significant, with a notably higher pica prevalence in Africa (*p* < 0.0001). Subgroup differences across study designs were insignificant (*p* = 0.7534).

#### 3.3.2. Materials Ingested

Five studies reported materials ingested by patients with pica [3, 4, 13, 19, 20, 24], although one study provided insufficient detail for data pooling [13]. This study from Rodrigues reported paper (*n* = 30), fabric (*n* = 13) and clay/dirt (*n* = 9) as the most ingested materials [13]. Data from the remaining four studies (*n* = 280) were pooled (Table 2), showing clay/dirt (*n* = 110, 39.3%), paper (*n* = 60, 21.4%) and fabric (*n* = 29, 10.4%) as the most common materials. The high clay/dirt count was largely driven by the Sudan study, in which 90 patients consumed clay [4].

**TABLE 2:** Materials ingested in 280 patients with sickle cell disease and pica.

Substance	Number of patients^∗^ *n* (%)
Clay or dirt	110 (39.3)
Paper (including tissue paper, toilet paper, paper towels, cardboard)	60 (21.4)
Fabric	29 (10.4)
Powder (including baby powder, cleansers and ashes)	18 (6.4)
Starch	16 (5.7)
Foam (including sponge, styrofoam and cushion foam)	12 (4.3)
Pencils	9 (3.2)
Coal	8 (2.9)
Limestone	6 (2.1)
Nails	5 (1.8)
Soap	5 (1.8)
Hair	4 (1.4)
Plastic	3 (1.1)
Toys	3 (1.1)
Gum	2 (0.7)
Erasers	2 (0.7)
Sand	2 (0.7)
Chalk	1 (0.4)
Jewel	1 (0.4)
Crayons	1 (0.4)
Popsicle sticks	1 (0.4)
Fabric closures (Velcro)	1 (0.4)

*Note:* The table summarizes the types of non‐nutritive, non‐food substances reported in patients with SCD and pica across included studies. Data from individual studies were pooled to present the frequency of each material ingested.

^∗^The sum of the number of patients exceeds 280 as some patients consumed two or three materials.

#### 3.3.3. Correlates

##### 3.3.3.1. Biological Correlates

###### 3.3.3.1.1. Age

Four studies directly investigated the relationship between age and pica in SCD. All four studies found an association between younger age and pica in SCD.

Ahmed, et al. found that pica was particularly prevalent in children between the ages of 6 and 8 years, in both an SCD group and control group. However, they did not directly compare the age of study inclusion between children with and without pica [4]. Aloni et al. reported that the median age of SCD patients with pica (7.4 years; range: 5.4–10.7) was significantly lower than that of those without pica (9.3 years; range: 7.0–12.3; *p* < 0.05) [19]. Accordingly, Ivascu et al. found that children with pica were significantly younger compared to those without pica (mean 7.9 ± 3.7 vs. 10.1 ± 4.0 years, *p* ≤ 0.001). This age difference persisted when stratifying by SCD genotype differentiating between HbSS/HbSβ0 (generally severe disease phenotype) and HbSC/HbSβ+ groups (generally less severe disease phenotype). More specifically, in the HbSS/HbSβ0 group, pica patients were 1.9 years younger on average (*M* = 8.5 vs. 10.4 years; *p* ≤ 0.001), and in the HbSC/HbSβ^+^ group, the difference was 3.1 years *M* = 6.6 vs. 9.7 years; *p* ≤ 0.001) [20]. Lastly, Lemanek et al. identified significant correlations between children’s age and pica, when using the Children’s Eating Behaviour Inventory (CEBI) including pica‐related items to measure dysfunctional eating/pica [21].

In contrast, descriptive data from Rodrigues’s mixed‐method analysis of pica in SCD and ages between patients with pica (*M* = 10.16 years ± 4.02 SD) and without pica (*M* = 9.86 years ± 3.87 SD) seemed similar, although no comparative analysis was undertaken to directly explore the relationship [13].

###### 3.3.3.1.2. Sex

Four studies examined the relationship between sex and pica in individuals with SCD, of which only two studies performed by Ivascu et al. and Lemanek et al. performed statistical comparisons. Ivascu et al. reported an almost equal sex distribution among patients with pica: 49.6% females and 50.4% males [20]. Moreover, Lemanek et al. reported that sex was not associated with dysfunctional eating behaviour, including pica [21].

Two studies provided only descriptive data. First, Rodrigues showed similar distributions with 53.4% males in the pica group and 49.1% males in the non‐pica group [13]. Second, Redding‐Lallinger et al. reported that pica occurred more frequently in females than in males (60% versus 40%) [25].

###### 3.3.3.1.3. SCD Genotype

Five studies reported data on the relationship between SCD genotype and pica, but only three performed statistical comparisons [20, 21, 26]. Ivascu et al. found a higher prevalence of pica in HbSS patients (35.7%) versus HbSC/HbSβ^+^ (25.5%, *p* = 0.03) [20]. Contrastingly, Lemanek et al. found no correlation between genotype and dysfunctional eating, including pica, based on both parent and child reports [21]. Similarly, Viswanathan et al. reported similar proportion of patients having pica in the HbSS/HbSβ0 groups (32/108 = 29.6%) and HbSC/HbSβ+ groups (19/71, 26.8%, *p* = 0.7). The authors did, however, report that spontaneous resolution was observed more often in patients with HbSC/B+ (13/19 = 68.4%) genotypes compared to patients with HbSS/HbSβ0 genotypes who were not on hydroxyurea or regular transfusion therapy and pica (6/21 = 28.6%, *p* = 0.01) [26]. Descriptive data from Rodrigues showed slightly fewer HbSS patients in the pica group (69.0%) than in the non‐pica group (76.4%), opposite to Ivascu et al. [13]. Furthermore, Redding‐Lallinger et al. also reported similar SCD genotype proportions between pica and non‐pica patients, though it remains unclear whether statistical tests were performed [25].

###### 3.3.3.1.4. G6PD Deficiency

One study investigated the association between glucose‐6‐phosphate dehydrogenase (G6PD) deficiency, an X‐linked acute haemolytic disease, and pica in SCD and found no significant association between pica and G6PD deficiency (OR = 4.15, 95% CI 0.62 – 27.72, *p* = 0.148), though the small sample (pica *n* = 31, non‐pica *n* = 24) may have limited statistical power [19].

###### 3.3.3.1.5. Bodyweight, Height, and BMI

Four studies examined the association between anthropometric measures (bodyweight, height and BMI), of which two studies focused on bodyweight and height. Ahmed et al. found that children with pica had lower growth parameters with 64.7% below −3 SD for height (vs. 35.3% non‐pica, *p* ~ 0.001) and 66.9% below −3 SD for bodyweight (vs. 33.1% non‐pica, *p* ∼ 0.001) [4].

Ivascu et al. reported significantly lower bodyweight in patients with pica aged 5.0–9.9 years (*M* = 23.5 kg; SD = 1.3 for pica vs. *M* = 28.0 kg; SD = 1.6 for non‐pica) (*p* = 0.03) and 15.0–19.0 years (*M* = 54.6 kg; SD = 2.6 for pica vs. *M* = 65.1 kg; SD = 3.5 for non‐pica, *p* = 0.02), though results may be skewed by outliers in a small sample. Overall, patients with pica in their study had significantly lower bodyweight (*p* < 0.001), although this finding should be interpreted cautiously, as pica patients were on average 2.2 years younger than non‐pica patients. No significant differences in height were reported by Ivascu et al. [20].

Three studies investigated BMI in relation to pica. Aloni et al. found nearly identical BMI values between pica (*M* = 16.1 kg/m^2^, range 15.3–17.6) and non‐pica groups (*M* = 16.4 kg/m^2^, range 14.9–18.9) (*p* > 0.05) [4]. Similarly, Rodrigues reported no significant differences in BMI percentiles among children with current pica, past pica, or no history of pica (*p* > 0.05) [13]. In contrast, Redding‐Lallinger et al. reported lower BMI in adult males with current pica (20 vs. 23 kg/m^2^, *p* = 0.0002) [25].

###### 3.3.3.1.6. Hydroxyurea and/or Transfusion Therapy

Two studies examined the relationship between hydroxyurea and/or transfusion therapy and pica in SCD. Aloni et al. found no significant association between hydroxyurea use and pica (OR = 0.59, 95% CI: 0.16–2.18; *p* = 0.493) [19]. Viswanathan et al. reported that HbSS/HbSβ^0^ patients receiving hydroxyurea or transfusion therapy were more likely to show improvement or resolution of pica (60% vs. 21%; *p* = 0.04) compared to patients not receiving hydroxyurea or transfusion therapy, hypothetically due to increased foetal haemoglobin [26]. The authors did not report separate analyses for hydroxyurea and transfusion therapy, preventing further distinction between these treatment effects.

###### 3.3.3.1.7. Comorbidities

Comorbidities examined in SCD‐related pica include stroke and cerebral vasculopathy. Ivascu et al. reported that 34.6% of 26 patients with stroke had a history of pica, similar to 31.6% in the non‐stroke group [20]. Aloni et al. found no significant association between abnormal transcranial Doppler findings and pica (OR = 0.85, 95% CI 0.22–3.21; *p* = 1.00) [19]. However, the authors did not specify which cutoff values were used to classify TCD results as abnormal, limiting interpretability of this outcome.

###### 3.3.3.1.8. Measures of Disease Severity

Four studies examined the association between disease severity and pica in SCD, using measures such as frequency and intensity of painful VOCs, hospitalizations, emergency visits, and composite severity scores.

Lemanek et al. found correlations between caregiver‐reported (*p* < 0.05) and child‐reported (*p* < 0.01) pain frequency scores and scores on the Child Eating Behaviour Inventory (CEBI) including pica items, but not with scores on the Children’s Version of the Eating Attitude Test (CHEAT) including pica items. Average pain intensity scores during a crisis (rated between 1 and 10) showed no correlation with either measure. Remarkably, number of hospitalizations in the last year correlated with Pica CHEAT scores but not with Pica CEBI scores [21]. Ivascu et al. reported similar mean hospital days for pica versus non‐pica patients (3.08 vs. 3.21; *p* = 0.85) [20]. Rodrigues investigated emergency visits instead of hospitalizations and found comparable emergency visits across current pica (2.80 ± 2.71), past pica (2.32 ± 1.76) and never pica (1.97 ± 1.86; *p* > 0.05) [13]. Moreover, Viswanathan et al. observed no difference in pica prevalence between patients with mild versus moderate‐severe disease as classified using a composite severity score consisting of number of hospitalizations and transfusions (*p* = 0.85) [26].

###### 3.3.3.1.9. Haemoglobin Level

Five studies examined the relationship between haemoglobin (Hb) levels and pica in SCD. Ahmed et al. (HbSS only) reported lower Hb in the pica group (79.3 ± 18.3 g/L) compared to that in the non‐pica group (90.2 ± 28.0 g/L; *p* = 0.02) [4]. Aloni et al. also found lower median Hb in the pica group (8.3 g/dL) versus non‐pica (9.1 g/dL; *p* < 0.01) [19]. Ivascu et al. observed lower Hb in the HbSS pica group (7.6 ± 1.0 g/dL) compared to that in the HbSS non‐pica (8.2 ± 1.1 g/dL; *p* < 0.001), with a near‐significant trend in HbSC/HbSβ^+^ pica (10.7 ± 1.3 g/dL) versus non‐pica (11.1 ± 1.1 g/dL; *p* = 0.07) [20]. In contrast, Lemanek et al. found no correlation between Hb and CEBI or ChEAT pica scores [21], and Rodrigues reported similar number of anaemia episodes across groups: current pica (2.05 ± 1.55), past pica (2.05 ± 1.8) and never pica (1.85 ± 1.7; *p* > 0.05) [13].

###### 3.3.3.1.10. Haematocrit Levels

Lemanek et al. found no significant correlations between haematocrit and scores on the CEBI and ChEAT including pica items [21].

###### 3.3.3.1.11. Red Blood Cell (RBC) Counts

Haemoglobin, haematocrit, and RBC count are interrelated measures of blood oxygen‐carrying capacity, with lower RBC counts generally leading to decreases in both haemoglobin and haematocrit. The relationship between pica and RBC counts was investigated by Ivascu et al. No differences were found between pica and non‐pica groups overall, or when stratified by genotype, with comparable values in HbSS patients (2.7 ± 0.4 × 10^6^/μL for pica and 2.8 ± 0.5 × 10^6^/μL for non‐pica patients, *p* = 0.24) and in HbSC/HbSB + patients (4.4 ± 0.7 × 10^6^/μL for pica and 4.5 ± 0.5 × 10^6^/μL for non‐pica patients, *p* = 0.36) [20].

###### 3.3.3.1.12. Mean Corpuscular Volume (MCV)

Four studies investigated the relationship between MCV and pica in SCD, with no significant associations reported. Ahmed et al. found no difference between children with and without pica (78.3 ± 14.6 fL vs. 81.4 ± 9.1 fL) [4], and Aloni et al. reported similar MCV values (88.5 ± 9.5 fL vs. 83.7 ± 11.1 fL; *p* = 0.157) between children with and without pica [19]. Ivascu et al. also observed no differences overall or by genotype [20], and Lemanek et al. found no correlations between MCV and CEBI or ChEAT pica scores [21].

###### 3.3.3.1.13. Reticulocyte Count

Three studies examined the association between reticulocyte counts and pica in SCD, with varying findings. Ahmed et al. reported significantly higher counts in children with pica (10.3 ± 9.8% vs. 6.0 ± 6.4%; *p* = 0.002) [4]. Aloni et al. found slightly higher counts in the pica group (334.3 vs. 270.6 × 10^3^/μL) but not statistically significant (*p* = 0.288) [19]. Ivascu et al. observed no significant differences overall or by genotype [20].

###### 3.3.3.1.14. Lactate Dehydrogenase (LDH)

Aloni et al. assessed LDH levels in SCD patients and found no significant difference between the pica (942.5 UI/L) and non‐pica groups (925 UI/L; *p* > 0.05) [19].

###### 3.3.3.1.15. Foetal Haemoglobin

Ivascu et al. found no differences in HbF levels between patients with and without pica, both overall and by genotype (HbSS and HbSC/HbSβ^+^) [20]. Viswanathan et al. did not directly assess HbF and pica, but reported greater HbF increases in patients whose pica improved or resolved after hydroxyurea or transfusion therapy compared to those without improvement (19.8% vs. 4.6%; *p* = 0.06), excluding non‐adherent patients, although not statistically significant [26].

###### 3.3.3.1.16. Micronutrients: Zinc, Copper, and Lead

Two studies explored the association between zinc levels and pica in SCD. Ahmed et al. reported lower mean serum zinc in patients with pica (46.4 mg/dL, SD 8.6) compared to those without (51.8 mg/dL, SD 10.7; *p* ~ 0.001) [4], whereas Aloni et al. found no significant difference (76 μg/L vs. 77 μg/L; *p* > 0.05) [19].

Aloni et al. also measured copper and lead levels, finding no differences between pica and non‐pica groups (copper: 140.4 vs. 135.0 μg/dL; lead: 20.5 vs. 13.0 μg/dL; *p* > 0.05), with all values within normal ranges [19]. Additionally, Redding‐Lallinger et al. reported that patients with current pica were more likely to have a diagnosed nutritional deficiency compared to those without (39% vs. 14%; *p* = 0.04), though it was not specified which deficiencies were examined [25].

###### 3.3.3.1.17. Measures of Iron Deficiency and Metabolism

Three studies assessed iron status, including serum ferritin and serum iron. Aloni et al. found no association between ferritin and pica, with levels of 155 ng/mL (range 77–275) in patients with pica versus 122 ng/mL (range 87–188) in those without (*p* > 0.05) [19]. In contrast, Redding‐Lallinger et al. observed lower mean ferritin in patients with current pica compared to those without (374 vs. 1256 ng/mL; *p* = 0.0069), though none were iron deficient [25]. Ahmed et al. reported that all patients with pica in their study had normal serum iron levels, and conducted no further group comparisons [4].

Texeira et al. examined the relationship between dietary iron intake and pica and found no significant association [23].

###### 3.3.3.1.18. Omega 3 Fatty Acid Supplementation

Khan et al. studied the effects of 6 months of omega‐3 supplementation on pica and caloric intake in Saudi Arabian children with SCD. Before the intervention, 26 of 66 participants had pica; afterward, no cases were reported, and the daily caloric intake increased significantly regardless of age or sex [24].

##### 3.3.3.2. Psychological Correlates

###### 3.3.3.2.1. Abnormal Eating/Dysfunctional Eating Patterns

Two studies examined the link between pica and dysfunctional eating. Lemanek et al. classified pica severity as follows based on caregiver reports: mild or moderate (seldom/sometimes) and severe (often/always). Children with severe pica had higher overall dysfunctional eating scores on the CEBI compared to non‐pica controls, but no association was seen in children with mild to moderate pica, nor when dysfunctional eating was assessed using the ChEAT [21].

Rodrigues assessed dysfunctional eating using the CEBI in a mixed‐methods study and found no significant differences among controls (*M* = 8.68, SD = 6.99), children with a history of pica (*M* = 9.41, SD = 5.57) and current pica (*M* = 11.77, SD = 5.78), though scores were slightly higher in the current pica group [13].

###### 3.3.3.2.2. Impulsivity

Rodrigues assessed emotional impulsivity symptoms in children with SCD and current pica, a history of pica and no history of pica using the negative and positive urgency subscales of the UPPS Impulsive Behaviour Scale—parent‐report version (higher scores = greater impulsivity). Children with current pica had higher scores (*M* = 11.07, SD = 10.23) than those with past pica (*M* = 4.42, SD = 6.05) or no history of pica (*M* = 4.50, SD = 6.55; *p* < 0.05) [13].

##### 3.3.3.3. Social Correlates

###### 3.3.3.3.1. Family History

Two studies examined the relationship between positive family history and pica in SCD. Ahmed et al. performed their research in 154 children with SCD (study group) and 50 healthy controls. They report that 66.2% of the study group had pica compared to 40% in the control group, and that a family history of pica was significantly higher in the study group compared to that in the control group (63.6% versus 28%, respectively, *p* ∼ 0.001). However, the difference in family history between pica and non‐pica patients in the SCD group was not directly assessed, limiting conclusions on this association [4]. Redding‐Lallinger et al. reported that 75% of individuals with pica had a sibling with pica versus 34% of without pica (*p* = 0.007). Further descriptive data reported that 83% of individuals with current pica had observed a family member consume non‐food items versus 50% of those without current pica [25].

###### 3.3.3.3.2. Satisfaction With Family Situation

Rodrigues assessed family satisfaction using the family satisfaction subscale of the Family Adaptability and Cohesion Evaluation Scale (FACES), which measures perceived family functioning in areas such as stress management, conflict resolution, and interpersonal relationships (higher scores = greater satisfaction). Parents of children with current pica reported significantly lower satisfaction (*M* = 37.88, SD = 8.44) than those with past pica (*M* = 44.32, SD = 4.64) or no history of pica (*M* = 42.42, SD = 6.79; *p* < 0.05) [13].

###### 3.3.3.3.3. SES

One study conducted by Lemanek et al. directly assessed the association between pica and SES defined by maternal income and education levels and found that lower SES was significantly associated with frequency of pica symptoms reported by caregivers (*p* < 0.05) [21]. Rodrigues only provided descriptive data, reporting lower annual family incomes ($39,795.92, SD 29,667) in those with current pica compared to those without current pica ($46,489.36, SD 30,161) [13].

###### 3.3.3.3.4. Food Insecurity

Rodrigues assessed food insecurity using the six‐item short form of the Food Security Survey Module (scores 0–12, higher scores = greater food insecurity). Children with current pica had slightly higher scores (*M* = 2.16, SD = 1.87) than those with past pica (*M* = 1.32, SD = 0.78) or no history of pica (*M* = 1.39, SD = 1.15), though differences were not significant, possibly due to limited power to detect small effects [13].

## 4. Discussion

This systematic review and meta‐analysis aimed to evaluate the prevalence and characteristics of pica among individuals with SCD at 34% (REM 34%, 95% CI 24–46%, I2 = 94%, *p* < 0.0001), 11 times higher than in the general population [2]. Subgroup analyses showed no differences between retrospective and cross‐sectional designs but revealed significant variations based on geographical region in which the study was performed, with the highest prevalence of pica in SCD reported in Africa. High rates of pica in Africa are not unique to SCD; with the highest global prevalence of pica among pregnant women also reported in Africa [27] and a study among healthy adolescents in Sudan reporting that 30.7% exhibited pica behaviour [28]. These elevated rates may reflect underlying factors such as low SES, food insecurity, or nutritional deficiencies.

Clay/dirt, paper, and fabric were the most commonly consumed materials, though this pattern was driven by high clay consumption in the Sudanese study by Ahmed et al. [4]. In most other studies, paper was most frequently reported, possibly reflecting regional differences in the availability of materials. The specific appeal of paper in SCD remains unclear.

Our literature synthesis of biological factors showed that younger age, lower bodyweight, and lower haemoglobin levels among HbSS patients were associated with pica. Other factors such as HbSS genotype, low body height/BMI, frequent VOCs, hospitalizations, reticulocyte counts, zinc, ferritin, and low omega‐3 intake were suggested but inconsistently or insufficiently supported to draw conclusions, warranting further research. Additionally, interpretation of certain biomarkers such as MCV remains challenging in SCD due to the influence of coexisting conditions including iron deficiency, α‐thalassemia, and variation in HbF levels. Interestingly, iron deficiency, often linked to pica among both anaemic and non‐anaemic individuals without SCD [9], could not be associated with pica in SCD. Similarly, despite evidence that zinc deficiency and supplementation influence pica in other populations [29], a relationship between zinc deficiency/levels and pica in SCD could not be confirmed in this systematic review.

Psychological factors associated with pica in SCD include higher impulsivity, possibly suggesting impaired behavioural inhibition contributing to its prevalence. Evidence linking pica to dysfunctional eating remains inconsistent across studies and assessment tools. The association with impulsivity reflects findings in the general population, where pica is often associated with neurodevelopmental disorders such as ADHD, a condition already reported to be more prevalent in children with SCD [30]. Impaired inhibition and impulsivity may therefore represent key mechanisms in the development of pica in SCD. In other populations, pica has been linked to anxiety, obsessive compulsive disorder and stress [31, 32]. Although higher rates of anxiety and depression have been documented among children and adolescents with SCD [33], the relationship between these conditions and pica in this population remains unexplored and requires further study.

Social factors associated with pica in SCD in our literature syntheses include positive family history, lower SES and lower family satisfaction. Lower family satisfaction, as measured by FACES‐IV, reflects poorer cohesion, flexibility and overall family functioning, often arising from conflict, poor communication or role imbalances [34], which may indirectly reflect increased family stress, a risk factor for pica as proposed in the biopsychosocial model [14]. Rodrigues also reported slightly higher food insecurity in children with pica compared to controls, though this difference was not statistically significant, possibly due to limited power to detect small effects. Therefore, the role of food insecurity warrants further investigation [13].

To date, no studies have specifically investigated neurobiological factors in pica among patients with SCD. Pica has been observed in individuals with brain injuries or neurodevelopmental disorders, especially in adults with memory loss or lesions in brain regions involving memory, decision‐making and impulse control (e.g., middle temporal gyrus and anterior bilateral lobes) [35, 36]. In SCD, where brain injury can occur as overt stroke, silent infarcts or microvascular damage, such neurological changes could potentially contribute to pica behaviour [37]. However, the relationship between SCD‐related brain damage and pica remains unexplored. Notably, while brain damage often accumulates with age [38], pica typically resolves in most children as they grow older [39], suggesting that neurological damage alone is insufficient to explain its occurrence. Behavioural and psychosocial factors are therefore likely key contributors. Future research incorporating neuroimaging alongside biological, psychological and social measures could clarify the mechanisms underlying pica in SCD and help guide targeted interventions.

Findings of our systematic review and meta‐analysis should be interpreted in the light of its strengths and limitations. To our knowledge, our systematic review and meta‐analysis—performed in accordance to PRISMA guidelines (Supplement 4)—is the first to synthesize evidence on the prevalence, characteristics and correlates of pica in individuals with SCD. We conducted an extensive search across multiple databases, including full‐text articles and abstracts, but the overall evidence remains limited due to the small number of studies, most of which were of low quality. Despite their limitations, we have included all available studies due to the scarcity of data. A further limitation of our study is that associations between pica and potential correlates could only be synthesized narratively. Although several studies examined similar variables, the small number of available studies, differences in measures or instruments used and lack of detailed data precluded meaningful statistical pooling. Consequently, we were unable to conduct meta‐analyses for these correlates. Abstracts were also included in our analysis; while abstracts may rely on preliminary findings, their inclusion increased the amount of data available. This decision aimed to improve the reliability of our estimates and reduce publication bias. Notably, we did not assess publication bias directly, as funnel plots are not suitable for meta‐analyses of pooled prevalence rates [40]. Most included studies were conducted in the United States, limiting the generalizability of our findings to other regions such as Europe. Additionally, the study from Sudan [4], which reported a notably higher prevalence of pica, may have contributed to an overall higher pooled prevalence in our meta‐analysis. It is therefore possible that the true prevalence of pica in Western countries is lower. Due to the high pica prevalence in the Sudanese study—where clay and dirt were the most consumed substances—these materials became the most frequently reported overall when data from all studies were combined. In contrast, in most other included studies, paper was the most consumed substance. Consequently, the conclusion that clay/dirt is the most frequently consumed substance may be skewed by this regional effect and might not accurately reflect pica patterns in Western settings. Moreover, none of the included studies utilized a validated diagnostic tool for pica, such as the PARDI interview [41], although all cross‐sectional studies used similar screening questions to identify pica. High heterogeneity in study populations and data collection was observed. To address this, we used logit transformation, the inverse variance method, and a REM, though prevalence estimates may still not fully reflect reality.

One retrospective study by Neikirk et al. retrieved with our search strategy was excluded because it included children as young as 1 year old, an age at which pica behaviour is developmentally typical [3]. This study did examine associations with abnormal eating, neurodevelopmental disorders, age, sex, stroke, frequency of pain crises, hydroxyurea or chronic transfusion therapy, vitamin D deficiency, comorbidities (constipation or diarrhoea) and various laboratory measures (including haemoglobin, HbF, reticulocyte count and measures of iron deficiency). They only found associations between high fast‐food consumption and neurodevelopmental disorders and pica [3].

In conclusion, this systematic review and meta‐analysis estimated a pooled pica prevalence of 34% among children with SCD and identified several potential correlates of pica in SCD, including younger age, lower bodyweight, lower haemoglobin levels in HbSS patients, higher impulsivity, positive family history, lower SES and reduced family satisfaction. Further research is needed to clarify the role of biological factors such as SCD genotype, low bodyweight or BMI, disease severity (VOCs, hospitalizations), reticulocyte counts, zinc, ferritin and omega‐3 intake; psychological factors including dysfunctional eating, anxiety, OCD and stress; social factors such as food insecurity; and neuroimaging findings, in the occurrence of pica in SCD. Therefore, larger longitudinal cohorts, investigating a comprehensive range of these biological, (neuro)psychological and social factors, with concomitant neuroimaging data, are needed to gain deeper insights into the mechanisms underlying the increased susceptibility to pica in SCD and to develop targeted interventions and therapeutic approaches.

## Funding

This research received no specific grant from any funding agency in the public, commercial or not‐for‐profit sectors.

## Disclosure

All authors have read and approved the final version of the manuscript. M.H.C. had full access to all of the data in this study and takes complete responsibility for the integrity of the data and the accuracy of the data analysis.

## Conflicts of Interest

M.H.C.’s institution has received investigator‐initiated research and travel grants as well as speaker fees over the years from the Netherlands Organization for Scientific Research (NWO) and Netherlands National research Agenda (NWA), the Netherlands Organization for Health Research and Development (ZonMw), the Dutch Innovatiefonds Zorgverzekeraars, Baxter/Baxalta/Shire/Takeda, Pfizer, Bayer Schering Pharma, CSL Behring, Sobi Biogen, Novo Nordisk, Novartis and Nordic Pharma and for serving as a steering board member for Roche, Bayer and Novartis for which fees go to the Erasmus MC as an institution. The other authors declare no conflicts of interest.

## Supporting Information

Additional supporting information can be found online in the Supporting Information section.

## Supporting information


**Supporting Information** Supporting 1: Detailed search strategies for all databases searched, including Embase, Medline, Cochrane, Web of Science, PsycINFO and Google Scholar. Supporting 2: Adapted version of the Newcastle–Ottawa Scale for quality assessment of cross‐sectional studies. Supporting 3: Additional tables and figures, comprising an overview table of study characteristics (Table S3T1), subgroup analysis of pica prevalence by geographic region (Figure S3F1) and subgroup analysis of pica prevalence by study design (Figure S3F2). Supporting 4: PRISMA documentation, including PRISMA checklist for systematic review and meta‐analysis (Table S4T1) and PRISMA checklist for abstracts of systematic review and meta‐analysis (Table S4T2).

## Data Availability

The data that support the findings of this study are available from the corresponding author upon reasonable request.
