# Supplementary material for: Pica in Sickle Cell Disease: A Systematic Review and Meta‐Analysis
Source: Anemia. 2026 Aug 19;2026:5589148. doi: 10.1155/anem/5589148 (PMC13490914; doi:10.1155/anem/5589148)
Supplement: Supplementary file 1 [file ANEM-2026-5589148-s001.docx]

# Supplements

## Supplement 1: Search strategies

Embase

('sickle cell anemia'/exp OR (sickle OR ((hemoglobin* OR haemoglobin*) NEXT/1 (SC OR SD OR beta* OR SS OR s*)) OR homozygous-SS OR HbS):ab,ti,kw) AND (pica/exp OR (pica OR geophag* OR pagophag* OR trichophag* OR ((dirt OR soil OR ice OR foam OR trichobezoar OR sharp OR starch OR matches OR feces OR faeces OR vomit OR raw-potatoes OR earth OR soil OR clay OR glass OR stones OR mucus OR hair OR wool OR fiber OR fibre OR urine OR blood OR wood OR paper OR sponge*) NEAR/3 (eat*)) OR acuphagia* OR amylophagia* OR cautopyreiophagia* OR coniophagia* OR coprophagia* OR emetophagia* OR geomelophagia* OR geophagia* OR hyalophagia* OR lithophagia* OR mucophagia* OR pagophagia* OR plumbophagia* OR trichophagia* OR urophagia* OR hematophagia* OR xylophagia*):ab,ti,kw)

Medline

(exp Anemia, Sickle Cell/ OR (sickle OR ((hemoglobin* OR haemoglobin*) ADJ (SC OR SD OR beta* OR SS OR s*)) OR homozygous-SS OR HbS).ab,ti,kf.) AND (Pica/ OR (pica OR geophag* OR pagophag* OR trichophag* OR ((dirt OR soil OR ice OR foam OR trichobezoar OR sharp OR starch OR matches OR feces OR faeces OR vomit OR raw-potatoes OR earth OR soil OR clay OR glass OR stones OR mucus OR hair OR wool OR fiber OR fibre OR urine OR blood OR wood OR paper OR sponge*) ADJ3 (eat*)) OR acuphagia* OR amylophagia* OR cautopyreiophagia* OR coniophagia* OR coprophagia* OR emetophagia* OR geomelophagia* OR geophagia* OR hyalophagia* OR lithophagia* OR mucophagia* OR pagophagia* OR plumbophagia* OR trichophagia* OR urophagia* OR hematophagia* OR xylophagia*).ab,ti,kf.)

Cochrane

((sickle OR ((hemoglobin* OR haemoglobin*) NEXT/1 (SC OR SD OR beta* OR SS OR s*)) OR homozygous-SS OR HbS):ab,ti,kw) AND ((pica OR geophag* OR pagophag* OR trichophag* OR ((dirt OR soil OR ice OR foam OR trichobezoar OR sharp OR starch OR matches OR feces OR faeces OR vomit OR raw-potatoes OR earth OR soil OR clay OR glass OR stones OR mucus OR hair OR wool OR fiber OR fibre OR urine OR blood OR wood OR paper OR sponge*) NEAR/3 (eat*)) OR acuphagia* OR amylophagia* OR cautopyreiophagia* OR coniophagia* OR coprophagia* OR emetophagia* OR geomelophagia* OR geophagia* OR hyalophagia* OR lithophagia* OR mucophagia* OR pagophagia* OR plumbophagia* OR trichophagia* OR urophagia* OR hematophagia* OR xylophagia*):ab,ti,kw)

Web of Science

TS=(((sickle OR ((hemoglobin* OR haemoglobin*) NEAR/1 (SC OR SD OR beta* OR SS OR s)) OR homozygous-SS OR HbS)) AND ((pica OR geophag* OR pagophag* OR trichophag* OR ((dirt OR soil OR ice OR foam OR trichobezoar OR sharp OR starch OR matches OR feces OR faeces OR vomit OR raw-potatoes OR earth OR soil OR clay OR glass OR stones OR mucus OR hair OR wool OR fiber OR fibre OR urine OR blood OR wood OR paper OR sponge*) NEAR/2 (eat*)) OR acuphagia* OR amylophagia* OR cautopyreiophagia* OR coniophagia* OR coprophagia* OR emetophagia* OR geomelophagia* OR geophagia* OR hyalophagia* OR lithophagia* OR mucophagia* OR pagophagia* OR plumbophagia* OR trichophagia* OR urophagia* OR hematophagia* OR xylophagia*)))

PsycINFO

(Sickle Cell Disease/ OR (sickle OR ((hemoglobin* OR haemoglobin*) ADJ (SC OR SD OR beta* OR SS OR s*)) OR homozygous-SS OR HbS).ab,ti.) AND (Pica/ OR (pica OR geophag* OR pagophag* OR trichophag* OR ((dirt OR soil OR ice OR foam OR trichobezoar OR sharp OR starch OR matches OR feces OR faeces OR vomit OR raw-potatoes OR earth OR soil OR clay OR glass OR stones OR mucus OR hair OR wool OR fiber OR fibre OR urine OR blood OR wood OR paper OR sponge*) ADJ3 (eat*)) OR acuphagia* OR amylophagia* OR cautopyreiophagia* OR coniophagia* OR coprophagia* OR emetophagia* OR geomelophagia* OR geophagia* OR hyalophagia* OR lithophagia* OR mucophagia* OR pagophagia* OR plumbophagia* OR trichophagia* OR urophagia* OR hematophagia* OR xylophagia*).ab,ti.)

Google Scholar

"sickle cell anemia|anaemia" pica|geophagia|pagophagia|trichophagia|acuphagia|amylophagia|cautopyreiophagia|coniophagia|coprophagia|emetophagia|geomelophagia|geophagia|hyalophagia|lithophagia|mucophagia|pagophagia|plumbophagia|trichophagia

'sickle cell anemia|anaemia' pica|geophagia|pagophagia|trichophagia|acuphagia|amylophagia|cautopyreiophagia|coniophagia|coprophagia|emetophagia|geomelophagia|geophagia|hyalophagia|lithophagia|mucophagia|pagophagia|plumbophagia|trichophagia

## Supplement 2

**Newcastle-Ottawa Scale adapted for cross-sectional studies**

Selection (maximum 5 stars):

*1.	Representativeness of the sample:*

a) Truly representative of the average in the target population. * (all subjects or random sampling). 1 score
b) Somewhat representative of the average in the target group. * (non-random sampling). 1 score
c) Selected group of patients. 0 score
d) No description of the derivation of the included subjects. 0 score

*2.	Sample size:*

a) Justified and satisfactory (>50 patients included). *	1 score
b) Not justified (<50 patients included). 0 score
c) No information provided. 0 score

*3.	Non-respondents:*

a) Basic summary of non-respondent characteristics in sampling frame recorded, or summary of characteristics of the whole sample is reported (e.g. how many patients are treated at the centre in which the study is conducted?). * 1 score
b) No summary data on non-respondents. 0 score
c) No information provided. 0 score

4.	*Ascertainment of pica*

a) Pica was correctly defined (study population >2 years) and was the main outcome of interest in this study. **	2 scores
b) Pica was not the main outcome of interest, but its definition and measurement has been clearly described, and the study was conducted in children > 2 years or adults. *	1 score
c) Pica was the main outcome of interest, but its definition and/or measurement was not clearly described. 0 score

Comparability: (Maximum 2 stars)

*1.	Comparability of subjects in different outcome groups based on design or analysis. Confounding factors controlled.*

a) The study investigates potential confounders or correlates (e.g. age, sex). *	1 score
b) The study does not investigate potential confounders. 0 score

Outcome (3 points):

*1.	Assessment of outcome:*

a) Validated screening/surveillance tool (e.g. PARDI interview) **. 2 scores
b) Not validated screening tool, but the tool is available or described clearly for replication. *	1 score
c) Hospital records. *	1 score
d) Not described. 0 score

*2.	Statistical test:*

a) Statistical test used to analyse the data clearly described, appropriate and measures of association presented including confidence intervals and probability level (p value). *	1 score
b) Statistical test not appropriate, not described, or incomplete. 0 score

Classification of studies:

Very Good Studies: 9-10 points

Good Studies: 7-8 points

Satisfactory Studies: 5-6 points

Unsatisfactory Studies: 0 to 4 points

This scale has been adapted from the Newcastle-Ottawa Quality Assessment Scale for cohort studies to provide quality assessment of cross-sectional studies.

# Supplement 3

**Table S3T1: Characteristics of included studies**

| **Author & Year** | **Country** | **Sample Size** | **Genotypes** | **Mean Age at Inclusion in years ± SD, Range** | **Pica Prevalence, n (%)** | **Study Design and Pica Assessment** | **Correlates Investigated** | **Main Findings** |
| --- | --- | --- | --- | --- | --- | --- | --- | --- |
| Ahmed et al. (2015) ^[4]^ | Sudan | 154 | HbSS | Range 2–18 | 102 (66%) | Cross-sectional; self-composed pica questionnaire | Age, height, bodyweight, family history, Hb, MCV, reticulocyte count, serum iron, and zinc | Pica more common in younger children (6–8 years). Significantly associated with positive family history, lower body weight and height, lower Hb, lower zinc, and higher reticulocyte count; not associated with other correlates. |
| Aloni et al. (2015) ^[17]^ | Belgium | 55 | X | 7.4 years, 5.4–10.7 pica; 9.3 years, 7.0–12.3 non-pica | 16 (29%) | Cross-sectional; self-composed pica questionnaire | Age, BMI, cerebral vasculopathy, copper, ferritin, G6PD deficiency, Hb, hydroxyurea, LDH, lead, MCV, reticulocyte count, sex, and zinc. | Pica significantly associated with lower Hb and younger age; no associations with other correlates. |
| Ivascu et al. (2001) ^[18]^ | USA | 395 | HbSS, HbSC, HbSβ+ | 7.9 ± 3.7 pica; 10.1 ± 4.0 non-pica | 134 (34%) | Retrospective; chart review for pica and synonyms | Age, body height, body weight, genotype, Hb, HbF, hospitalisations, MCV, RBC, reticulocyte count, sex, stroke | Pica significantly associated with HbSS genotype, younger age, lower Hb, lower RBC, and lower body weight in HbSS group; no association with other correlates. |
| Khan et al. (2019) ^[24]^ | Saudi Arabia | 66 | X | Range 5–16 | 13 (20%) | Cross-sectional; method not reported | Omega-3 fatty acid intake. | No pica observed after omega-3 supplementation. |
| Lemanek et al. (2002) ^[19]^ | USA | 146 (75 M, 71 F) | HbSS (n=93), HbSC (n=43), HbSβ0 (n=3) | Mean age 7.6, ± 3.7, 2–16 | 91 (62%) | Cross-sectional; self-composed pica questionnaire | Age, genotype, Hb, haematocrit, hospitalisations, MCV, pain frequency/severity, SES (maternal income and education), and sex. | Pica associated with younger age and lower SES; no clear associations with other correlates. |
| Redding-Lallinger et al. (2005) ^[21]^ | USA | 86 | HbSS, HbSβ0, HbSC, HbSβ+ | Range 19–63 | 13 (16%) | Retrospective chart review | BMI, family history, ferritin, genotype, nutritional deficiency, osteopenia, sex. | Current pica more prevalent in females. Current pica associated with nutritional deficiency, lower BMI (males only), lower ferritin, higher positive family history; no difference in osteopenia. |
| Reed-Knight et al. (2015) ^[20]^ | USA | 176 | X | X | 49 (28%) | Cross-sectional; self-composed pica questionnaire | X | Pica identification rate 28% across three hospital sites; correlates not investigated. |
| Rodrigues (2019) ^[11]^ | USA | 113 | All SCD genotypes | 10.16 ± 4.02 pica; 9.86 ± 3.87 non-pica | 49 (28%) | Cross-sectional; self-composed pica questionnaire | Age, anemia, BMI, dysfunctional eating, ER visits, family income, family satisfaction, food insecurity, genotype, impulsivity, sex. | Lower family satisfaction and higher impulsivity associated with pica; no differences in anemia, BMI, ER visits, dysfunctional eating, or food insecurity. |
| Teixeira et al. (2023) ^[23]^ | Brazil | 215 | HbSS | Range 19–59 | 40 (18.6%) | Cross-sectional; self-composed pica questionnaire | Iron intake | No significant association between iron intake and pica. |
| Viswanathan et al. (2015) ^[22]^ | USA | 179 | HbSS/HbSβ0 (n=108), HbSβ+/HbSC (n=71) | Range 2–21 | 51 (29%) | Retrospective chart review | Disease severity, genotype, HbF improvement, hydroxyurea, regular transfusion therapy | No significant difference by genotype or disease severity. Lower pica prevalence with regular transfusions. Spontaneous resolution more common in HbSC/HbSB+. Resolution/improvement more frequent in HbSS/HbSβ0 on therapy and in those with higher HbF increase. |

Table S3T1: Overview of characteristics of included studies. The table summarizes key features of the studies included in the systematic review, including authors, year, country, study design, sample size, population characteristics, and main outcomes.

Abbreviations: BMI, body mass index; ER, emergency room; G6PD, glucose-6-phosphate dehydrogenase; Hb, hemoglobin; HbF, foetal hemoglobin; HbSβ0, sickle-beta-zero thalassemia; HbSβ+, sickle-beta-plus thalassemia; HbSC, sickle-hemoglobin C disease; HbSS, homozygous sickle cell anemia; LDH, lactate dehydrogenase; MCV, mean corpuscular volume; RBC, red blood cell count; SD, standard deviation; SES, socioeconomic status; vs, versus; X, not reported/not applicable.

**Figure S3F1: Subgroup analysis by geographic region**

Figure S3F1: Subgroup analysis by geographical region. Forest plot displaying proportion estimates of pica from individual studies and the pooled proportion, stratified by geographical region.

Squares represent study-specific proportion with 95% confidence intervals (horizontal lines), with square size proportional to study weight. Diamonds represent pooled proportion estimates within each region and overall.

**Figure S3F2: Subgroup analysis by study design**

Figure S3F2: Subgroup analysis by study design. Forest plot displaying proportion estimates of pica from individual studies and the pooled proportion, stratified by study design.

Squares represent study-specificproportion with 95% confidence intervals (horizontal lines), with square size proportional to study weight. Diamonds represent pooled proportion estimates within each study design category and overall.

# Supplement 4

**S4T1: Prisma checklist full text article**

| **Section and Topic** | **Item #** | **Checklist item** | **Location where item is reported** |
| --- | --- | --- | --- |
| **TITLE** | | |  |
| Title | 1 | Identify the report as a systematic review. | Title |
| **ABSTRACT** | | |  |
| Abstract | 2 | See the PRISMA 2020 for Abstracts checklist. | See PRISMA abstract checklist |
| **INTRODUCTION** | | |  |
| Rationale | 3 | Describe the rationale for the review in the context of existing knowledge. | Introduction (page 2-3) |
| Objectives | 4 | Provide an explicit statement of the objective(s) or question(s) the review addresses. | Introduction (page 3) |
| **METHODS** | | |  |
| Eligibility criteria | 5 | Specify the inclusion and exclusion criteria for the review and how studies were grouped for the syntheses. | Methods (page 3) |
| Information sources | 6 | Specify all databases, registers, websites, organisations, reference lists and other sources searched or consulted to identify studies. Specify the date when each source was last searched or consulted. | Methods (page 3) |
| Search strategy | 7 | Present the full search strategies for all databases, registers and websites, including any filters and limits used. | Supplement 1 |
| Selection process | 8 | Specify the methods used to decide whether a study met the inclusion criteria of the review, including how many reviewers screened each record and each report retrieved, whether they worked independently, and if applicable, details of automation tools used in the process. | Methods (page 3) |
| Data collection process | 9 | Specify the methods used to collect data from reports, including how many reviewers collected data from each report, whether they worked independently, any processes for obtaining or confirming data from study investigators, and if applicable, details of automation tools used in the process. | Methods (page 3) |
| Data items | 10a | List and define all outcomes for which data were sought. Specify whether all results that were compatible with each outcome domain in each study were sought (e.g. for all measures, time points, analyses), and if not, the methods used to decide which results to collect. | Methods (page 3) |
|  | 10b | List and define all other variables for which data were sought (e.g. participant and intervention characteristics, funding sources). Describe any assumptions made about any missing or unclear information. | Methods (page 3) |
| Study risk of bias assessment | 11 | Specify the methods used to assess risk of bias in the included studies, including details of the tool(s) used, how many reviewers assessed each study and whether they worked independently, and if applicable, details of automation tools used in the process. | Methods (page 4) |
| Effect measures | 12 | Specify for each outcome the effect measure(s) (e.g. risk ratio, mean difference) used in the synthesis or presentation of results. | Methods (page 4) |
| Synthesis methods | 13a | Describe the processes used to decide which studies were eligible for each synthesis (e.g. tabulating the study intervention characteristics and comparing against the planned groups for each synthesis (item #5)). | Methods (page 4) |
|  | 13b | Describe any methods required to prepare the data for presentation or synthesis, such as handling of missing summary statistics, or data conversions. | Methods (page 4) |
|  | 13c | Describe any methods used to tabulate or visually display results of individual studies and syntheses. | Methods (page 4) |
|  | 13d | Describe any methods used to synthesize results and provide a rationale for the choice(s). If meta-analysis was performed, describe the model(s), method(s) to identify the presence and extent of statistical heterogeneity, and software package(s) used. | Methods (page 4) |
|  | 13e | Describe any methods used to explore possible causes of heterogeneity among study results (e.g. subgroup analysis, meta-regression). | Methods (page 4) |
|  | 13f | Describe any sensitivity analyses conducted to assess robustness of the synthesized results. | Methods (page 4) |
| Reporting bias assessment | 14 | Describe any methods used to assess risk of bias due to missing results in a synthesis (arising from reporting biases). | Not performed (see discussion) |
| Certainty assessment | 15 | Describe any methods used to assess certainty (or confidence) in the body of evidence for an outcome. | Not performed |
| **RESULTS** | | |  |
| Study selection | 16a | Describe the results of the search and selection process, from the number of records identified in the search to the number of studies included in the review, ideally using a flow diagram. | Results (page 5) |
|  | 16b | Cite studies that might appear to meet the inclusion criteria, but which were excluded, and explain why they were excluded. | N/A |
| Study characteristics | 17 | Cite each included study and present its characteristics. | Supplement 2 (Table S3T1) |
| Risk of bias in studies | 18 | Present assessments of risk of bias for each included study. | Results (page 6): Table 1 |
| Results of individual studies | 19 | For all outcomes, present, for each study: (a) summary statistics for each group (where appropriate) and (b) an effect estimate and its precision (e.g. confidence/credible interval), ideally using structured tables or plots. | Results page 7 & Supplement 3F1 t/m F2 |
| Results of syntheses | 20a | For each synthesis, briefly summarise the characteristics and risk of bias among contributing studies. | Results (page 6) |
|  | 20b | Present results of all statistical syntheses conducted. If meta-analysis was done, present for each the summary estimate and its precision (e.g. confidence/credible interval) and measures of statistical heterogeneity. If comparing groups, describe the direction of the effect. | Results (page 7) |
|  | 20c | Present results of all investigations of possible causes of heterogeneity among study results. | Results (page 7) |
|  | 20d | Present results of all sensitivity analyses conducted to assess the robustness of the synthesized results. | Results (page 7) |
| Reporting biases | 21 | Present assessments of risk of bias due to missing results (arising from reporting biases) for each synthesis assessed. | Not assessed |
| Certainty of evidence | 22 | Present assessments of certainty (or confidence) in the body of evidence for each outcome assessed. | Discussion |
| **DISCUSSION** | | |  |
| Discussion | 23a | Provide a general interpretation of the results in the context of other evidence. | Discussion (page 16) |
|  | 23b | Discuss any limitations of the evidence included in the review. | Discussion (page 17-18) |
|  | 23c | Discuss any limitations of the review processes used. | Discussion (page 17-18) |
|  | 23d | Discuss implications of the results for practice, policy, and future research. | Discussion (page 18) |
| **OTHER INFORMATION** | | |  |
| Registration and protocol | 24a | Provide registration information for the review, including register name and registration number, or state that the review was not registered. | Review was not registered |
|  | 24b | Indicate where the review protocol can be accessed, or state that a protocol was not prepared. | Protocol was not prepared |
|  | 24c | Describe and explain any amendments to information provided at registration or in the protocol. | N/A |
| Support | 25 | Describe sources of financial or non-financial support for the review, and the role of the funders or sponsors in the review. | This review did not receive any (non-)financial support. |
| Competing interests | 26 | Declare any competing interests of review authors. | Declaration of interest statement |
| Availability of data, code and other materials | 27 | Report which of the following are publicly available and where they can be found template data collection forms; data extracted from included studies; data used for all analyses; analytic code; any other materials used in the review. | Data collection forms and extracted data available upon request |

Table S4T1: Prisma 2020 checklist for systematic review and meta-analysis. The table presents the items recommended by the PRISMA 2020 guidelines, indicating how each item was addressed in this review, including study selection, data extraction, risk of bias assessment, and synthesis methods.
From:  Page MJ, McKenzie JE, Bossuyt PM, Boutron I, Hoffmann TC, Mulrow CD, et al. The PRISMA 2020 statement: an updated guideline for reporting systematic reviews. BMJ 2021;372:n71. doi: 10.1136/bmj.n71. This work is licensed under CC BY 4.0. To view a copy of this license, visit <https://creativecommons.org/licenses/by/4.0/>

**S4T2: PRISMA abstract checklist**

| **Section and Topic** | **Item #** | **Checklist item** | **Reported (Yes/No)** |
| --- | --- | --- | --- |
| **TITLE** | | |  |
| Title | 1 | Identify the report as a systematic review. | Yes |
| **BACKGROUND** | | |  |
| Objectives | 2 | Provide an explicit statement of the main objective(s) or question(s) the review addresses. | Yes |
| **METHODS** | | |  |
| Eligibility criteria | 3 | Specify the inclusion and exclusion criteria for the review. | Yes |
| Information sources | 4 | Specify the information sources (e.g. databases, registers) used to identify studies and the date when each was last searched. | Yes |
| Risk of bias | 5 | Specify the methods used to assess risk of bias in the included studies. | Yes |
| Synthesis of results | 6 | Specify the methods used to present and synthesise results. | Yes |
| **RESULTS** | | |  |
| Included studies | 7 | Give the total number of included studies and participants and summarise relevant characteristics of studies. | Yes |
| Synthesis of results | 8 | Present results for main outcomes, preferably indicating the number of included studies and participants for each. If meta-analysis was done, report the summary estimate and confidence/credible interval. If comparing groups, indicate the direction of the effect (i.e. which group is favoured). | Yes |
| **DISCUSSION** | | |  |
| Limitations of evidence | 9 | Provide a brief summary of the limitations of the evidence included in the review (e.g. study risk of bias, inconsistency and imprecision). | Yes |
| Interpretation | 10 | Provide a general interpretation of the results and important implications. | Yes |
| **OTHER** | | |  |
| Funding | 11 | Specify the primary source of funding for the review. | N/A |
| Registration | 12 | Provide the register name and registration number. | N/A |

*Table S4T2: Prisma 2020 checklist for systematic review and meta-analysis abstracts.* The table summarizes the PRISMA 2020 items recommended for reporting in abstracts of systematic reviews and meta-analyses, indicating how each item was addressed in this review’s abstract.
From:  Page MJ, McKenzie JE, Bossuyt PM, Boutron I, Hoffmann TC, Mulrow CD, et al. The PRISMA 2020 statement: an updated guideline for reporting systematic reviews. BMJ 2021;372:n71. doi: 10.1136/bmj.n71. This work is licensed under CC BY 4.0. To view a copy of this license, visit <https://creativecommons.org/licenses/by/4.0/>
